# Environmental Amoxicillin Exposure Induces Cardiotoxicity in Zebrafish Embryos: A Comprehensive Assessment of Heart Function and Molecular Responses

**DOI:** 10.3390/antibiotics14101029

**Published:** 2025-10-14

**Authors:** Azza Naïja, Dalal Mohamed, Somaiya Abdulhakim, Amera Mohamed, Huseyin Cagatay Yalcin

**Affiliations:** 1 Biomedical Research Center, QU Health, Qatar University, Doha P.O. Box 2713, Qatar; anaija@qu.edu.qa (A.N.); dalalomar298@gmail.com (D.M.); 2 Biomedical Science Department, College of Health Sciences, QU Health, Qatar University, Doha P.O. Box 2713, Qatar; 3 Department of Mechanical and Industrial Engineering, Qatar University, Doha P.O. Box 2713, Qatar

**Keywords:** pharmaceuticals, amoxicillin (AMX), *Danio rerio*, cardiotoxicity, gene expression

## Abstract

**Background/Objectives.** Environmental pollution poses a significant threat to human health, primarily through the degradation of natural ecosystems. Emerging organic contaminants (EOCs), such as pharmaceuticals like amoxicillin, are especially concerning due to their persistence and potential harm to non-target species. This study evaluates the cardiotoxic effects of Amoxicillin (AMX) on Zebrafish embryos (*Danio rerio*), specifically heart function, blood flow, and gene expression linked to cardiogenesis, inflammation, and apoptosis. **Methods.** Zebrafish embryos were exposed to concentrations of AMX corresponding to environmentally relevant levels, wastewater effluents, as well as acute experimental exposures. Mortality and hatching rates were all assessed, along with heart function and gene expression analysis of key cardiac and inflammatory markers. **Results/Conclusions.** The findings suggest that higher AMX concentrations have cardiotoxic effects, emphasizing the need for stringent environmental monitoring of antibiotic residuals.

## 1. Introduction

Pharmaceutical pollution has emerged as a pressing environmental concern, with antibiotics representing one of the most frequently detected and environmentally persistent classes of contaminants in aquatic systems [1,2]. Residues from hospital effluents, agricultural runoff, and wastewater treatment plants have been consistently reported in rivers, lakes, and sediments, where they disrupt microbial communities and accelerate the spread of antibiotic resistance [3,4]. Antibiotics are of particular concern due to their biological activity and potential to exert effects on non-target organisms, even at trace concentrations [2,5].

The global consumption of antibiotics has been steadily increasing, with their extensive use as therapeutic agents in both human and veterinary medicine. Over the past few decades, this usage has expanded exponentially, driven by population growth, intensive farming practices, and advances in medical care. According to a recent World Health Organization (WHO) study, the median value of the overall consumption of antibacterial agents is 16.6 Defined Daily Dose (DDD) per 1000 inhabitants per day [6]. Although most antibiotics have a pharmacological half-life of only a few hours to several days in humans and animals, their residues are considered ‘pseudo-persistent’ organic contaminants in the environment.

Among antibiotics, AMX is a semisynthetic β-lactam and one of the most prescribed worldwide. It is used to treat and prevent bacterial infections in humans and animals. In 2022, the World Health Organization (WHO) reported that AMX was the most frequently consumed oral antibiotic (19% median relative consumption), ranking #1 in 12 (46%) of the 26 countries and territories that submitted data, and being in the DU75 of 24 countries and territories.

As a result of its high excretion in an unmetabolized (80–90% unchanged), AMX is frequently detected in aquatic environments, with concentrations up to 11.99 μg/L in surface waters and effluents [7]. While the ecotoxicological focus has often been on antibiotic resistance, a growing body of evidence indicates that antibiotics like AMX can have direct toxic effects on aquatic fauna [1,8]. Beyond its antimicrobial action, AMX exposure has been associated with systemic effects, including cardiovascular and neurological impacts in animal and clinical models [9,10]. For example, studies have demonstrated that AMX can have adverse effects on multiple organs, with evidence of cardiovascular and neurological impacts in animal and clinical models. Ref. [9] reported that early-life exposure to AMX in rats led to altered gut microbiota and reduced blood pressure, which may indicate systemic and cardiovascular effects. Moreover, in clinical settings, prolonged AMX use has been associated with rare but reported cases of neurotoxicity and hepatotoxicity [10].

Zebrafish (*Danio rerio*) are widely used in toxicological and developmental studies because of their rapid embryogenesis, optical transparency, and genetic tractability [11,12,13]. Importantly, zebrafish share ~70% of human genes, including highly conserved cardiac regulators such as *gata4/5/6*, *tbx5*, and *tbx20* [14,15]. These similarities make zebrafish embryos particularly suitable for cardiotoxicity research, enabling real-time visualization of heart morphology, hemodynamics, and molecular pathways relevant to human cardiovascular health [16]. However, prior zebrafish studies on AMX have largely examined genotoxicity, developmental delays, and biochemical responses [17,18], but comprehensive assessments of cardiac function and gene expression linked to cardiogenesis, apoptosis, and inflammation remain scarce. This knowledge gap motivated the present study, which aimed to provide an in-depth evaluation of AMX-induced cardiotoxicity in zebrafish embryos using functional and molecular endpoints.

## 2. Results

### 2.1. Embryotoxicity Assessment

**Figure 1:**
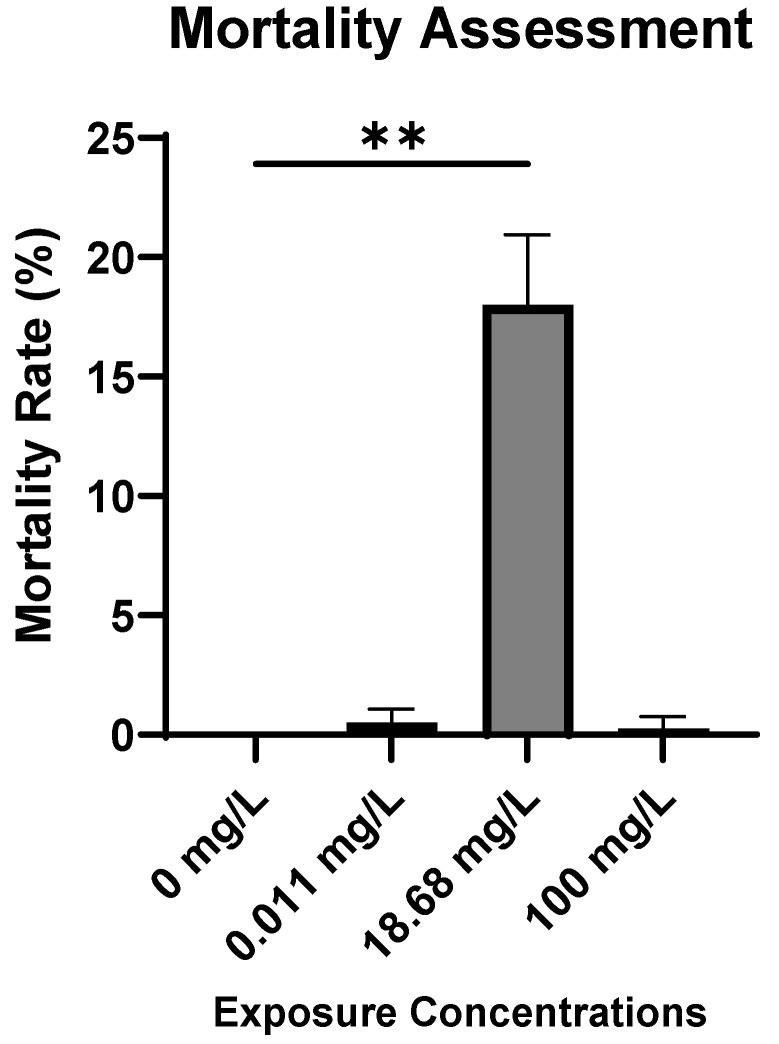
Mortality Rate (%) in AMX-exposed embryos at different exposure concentrations. Results are represented as mean ± SD (Kruskal–Wallis test: ** *p* < 0.01).

**Mortality Assessment.** Mortality rates in AMX-exposed embryos were determined at 96 hpf as shown in Figure 1. No mortality was observed in the control group throughout the experiment. Our findings revealed that the highest mortality rate among embryos occurred at the intermediate concentration (C2) of AMX, resulting in the mortality of 18.8% of the tested embryos.

**Figure 2:**
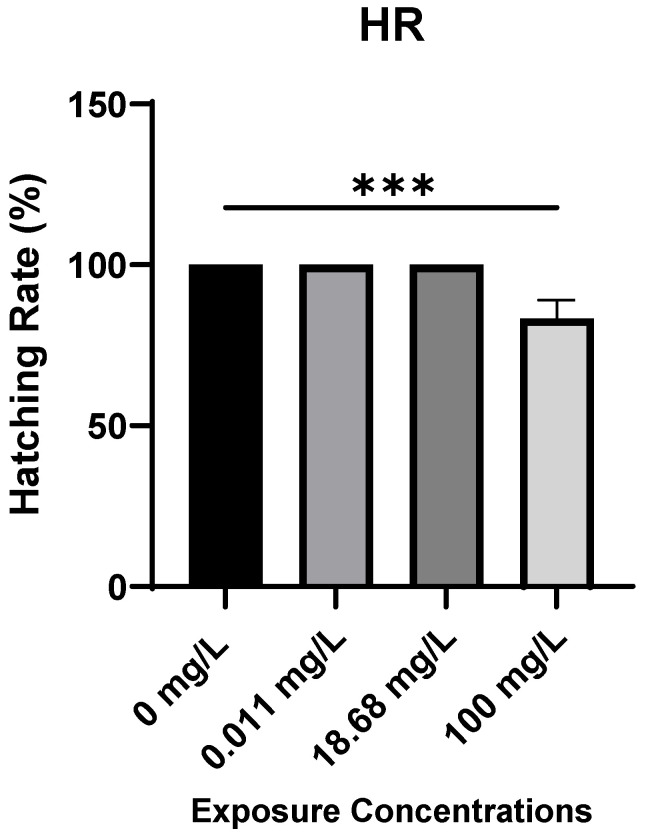
Hatching rate (%) of Zebrafish embryos at different time points of the experiment. Results are represented as mean ± SD (One-Way ANOVA test: *** *p* < 0.001).

**Hatching Rates.** The hatching rate (HR) of zebrafish embryos at 96 hpf showed a significant decrease with increasing AMX concentration (Figure 2). Embryos in the control group (0 mg/L) and those exposed to 0.011 mg/L and 18.9 mg/L exhibited nearly complete hatching (>95%) by 96 hpf. However, embryos exposed to 100 mg/L displayed a noticeable reduction in HR (approximately 80%), which was significantly lower compared to the control group (*p* < 0.001). These findings indicate that high concentrations of AMX can delay or impair hatching in zebrafish embryos.

### 2.2. Gene Expression

Figure 1 represents the relative expression profiles of major cardiac gene biomarkers (*gata4*, *gata5*, *gata6*, *tbx5*, *tbx20*, *bcl2*, *casp9*, and *il6*) in zebrafish embryos exposed to AMX at different concentrations. The selected AMX concentrations were determined based on amounts measured in the environment (C1), treatment plant effluents (C2), and acute exposures (C3). The reference gene used is *ef1a*, which is widely recommended for normalization in zebrafish developmental and toxicogenomic studies due to its stable expression across various experimental conditions [19,20]. All results are presented below under three thematic categories: Developmental Effects, Apoptotic Markers, and Inflammation. Throughout the experiment, the selected gene expressions remained stable and showed no significant changes in the control groups. AMX-exposed embryos showed varied expressions of the targeted genes. AMX exposure resulted in notable changes in the expression of all genes over the exposure period.

**Figure 3:**
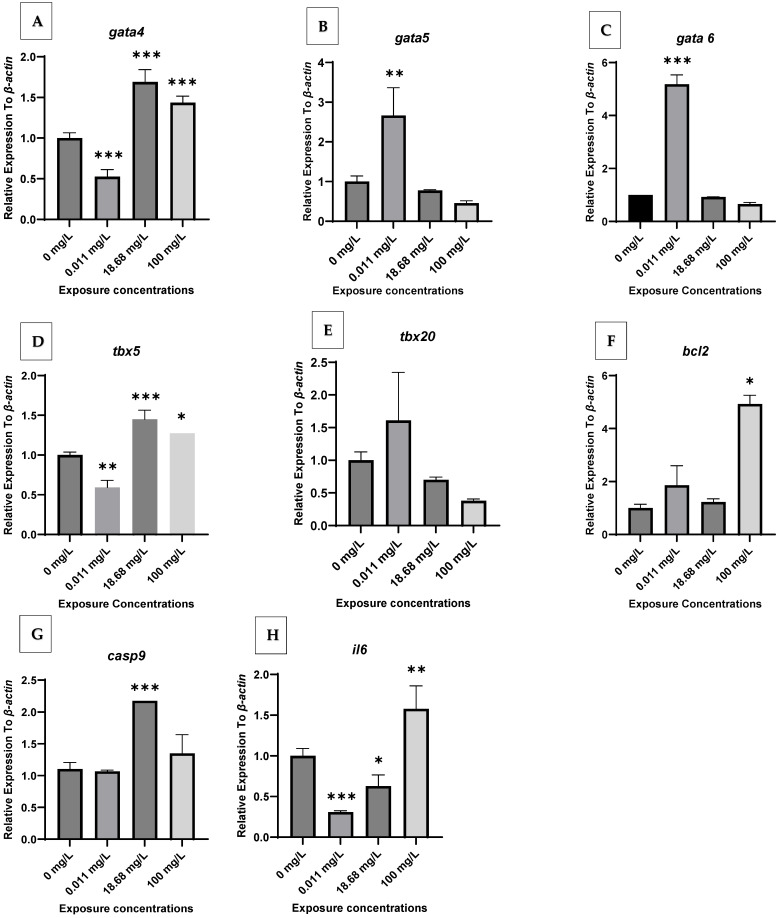
Relative Expression to *ef1a* in Zebrafish embryo controls and AMX-exposed groups at different exposure concentrations for *gata4* (**A**), *gata5* (**B**), *gata6* (**C**), *tbx5* (**D**), *tbx20* (**E**), *bcl2* (**F**), *casp9* (**G**), and *il6* (**H**). (Controls: 0 mg/L). (C1: 0.011 mg/L, C2: 18.68 mg/L, C3: 100 mg/L of AMX). Each bar represents mean ± SD. * denotes significant differences to controls for a given exposure concentration (Dunnett’s test, * *p* < 0.05, ** *p* < 0.01, *** *p* < 0.001).

*Developmental Effects (gata4, gata5, gata6, tbx5, and tbx20).* AMX exposure increased *gata4* gene expression in treatment groups C2 and C3, with C2 (18.68 mg/L) causing the highest *gata4* expression; however, treatment group C1 showed a significant decrease in gene expression (Figure 3A). For the gene *gata5*, mRNA expression in C2 and C3 were not significantly different from the NC group; however, expression in treatment group C1 (0.011 mg/L) was upregulated (Figure 3A). Similarly, *gata6* and *tbx20* followed the same trend as *gata5* (Figure 3C,E). The *tbx5* gene expression was highest in C2 and C3, whereas C1 had lower expression levels (Figure 3D).

*Apoptotic Markers (bcl2, casp9).* The expression of the gene *bcl2* was significantly reduced in treatment group C1, and the expression was not significant in the treatment group C3, while the expression in treatment group C2 was similar to the negative control (Figure 3F). *casp9* gene expression was upregulated in response to C2 and C3 concentrations, while the gene was expressed similarly to the negative control in treatment group C1 (Figure 3G).

*Inflammation (il6). il6* was downregulated in AMX-treatment groups C1 and C2 except for treatment group C3 (Figure 3H). These findings indicate that AXM exposure has a moderate influence on the cardiac system but a greater effect on the inflammatory pathway.

It is noteworthy that some genes, particularly *gata4* and *bcl2*, exhibited non-linear or non-monotonic dose–response patterns, with stronger effects at lower concentrations. This trend may reflect AMX instability in aqueous solutions or compensatory mechanisms in zebrafish embryos.

### 2.3. Heart Function Analysis

**Figure 4:**
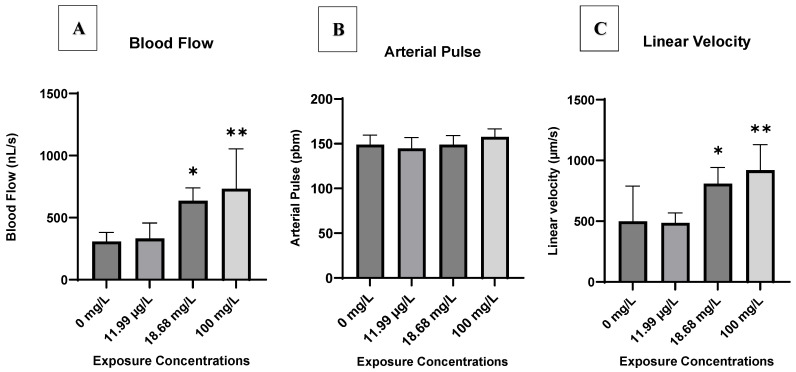
Heart function analysis in PCV of Zebrafish control and AMX-exposed at 96 hpf. Blood flow (**A**), arterial pulse (**B**), linear velocity (**C**). C1: 0.011 mg/L, C2: 18.68 mg/L, C3: 100 mg/L of AMX) Each bar represents mean ± SD. * denotes significant differences to controls for a given exposure concentration (Dunnett’s test, * *p* < 0.05, ** *p* < 0.01).

The results pertaining to zebrafish embryo heart function measured in the Post Cardinal Vein (PCV) are presented in Figure 4. Throughout the entire exposure duration to AMX, blood flow, arterial pulse, and linear velocity of control embryos remained consistent in the PCV, exhibiting no observable changes (*p* > 0.05). Analysis of the blood flow and linear velocity in AMX-exposed group hearts revealed that AMX affects both parameters in the PCV of AMX-treated embryos. A significant effect of AMX on blood flow and linear velocity in the PCV was observed in treated groups C2 and C3, with the highest effect at the exposure concentration of 100 mg AMX/L in treated group C3 (Figure 4A,C). However, exposure to AMX did not induce significant changes in arterial pulse in the PCV (Figure 4B).

## 3. Discussion

In the present study, we investigated the potential cardiotoxic effect of AMX using zebrafish embryos. The selected AMX concentrations were determined based on amounts measured in the environment (C1), sewage treatment plant effluents (C2), and acute exposures (C3).

***AMX-exposure concentration did not reach LC50 at 96 hpf***. Our toxicity tests demonstrated that AMX has a relatively low lethal effect on zebrafish embryos, with mortality rates not exceeding 18.8% at 96 hpf. The highest mortality occurred at 100 mg/L (C3), corresponding to LC18, while lower doses (11.99 and 18.68 mg/L) did not induce any mortality. A similar study also reported that AMX at environmentally relevant concentrations has minimal lethal effects on exposed fish [17].

The absence of a consistent dose–response relationship, with no mortality at lower concentrations and only moderate mortality at the highest concentration, may be due to several factors. Although our study did not directly measure this, previous research suggests that precipitation of AMX or its adsorption to plastic surfaces at higher nominal doses may reduce the actual bioavailable fraction, potentially explaining the lack of a clear dose–response relationship [1,7]. In line with this, the observed mortality difference between groups was relatively minimal, with the highest mortality at 18.8% (C2), indicating only a slight variation in survival across exposure levels. Second, AMX instability in aqueous solutions—including hydrolysis and photodegradation—can alter the actual exposure levels during the test period, especially in relation to pH and ionic composition [4]. Finally, stage-specific sensitivity and compensatory mechanisms in zebrafish embryos may contribute to the observed non-monotonic pattern, which has been described for other pharmaceuticals and endocrine-active substances in fish embryos [2].

Future studies should include measured concentrations over time, additional replicates, and standardized physicochemical controls to confirm whether this pattern reflects true biological variability or experimental factors.

*High AMX concentrations reduced the hatching rate at 96 hpf*. The observed reduction in hatching rate at 100 mg/L AMX suggests that high concentrations of the antibiotic can interfere with embryonic development and chorion softening processes required for successful hatching. Several mechanisms may explain this effect. AMX exposure at elevated levels may alter enzymatic activity of hatching enzymes such as chorionase, which is essential for chorion digestion during hatching [17]. Additionally, osmotic and ionic imbalances caused by high antibiotic loads can affect water uptake, leading to delayed chorion rupture [21]. Furthermore, stress induced by high AMX concentrations could disrupt normal metabolic pathways, reducing energy availability for muscular contractions required for hatching [2]. Similar findings have been reported for other antibiotics and pharmaceutical contaminants, where elevated exposure levels delayed or inhibited hatching in zebrafish embryos [7,17].

***AMX affected the cardiogenesis and heart function in zebrafish embryos.*** In this study, we investigated the expression of genes involved in cardiac development (*gata4*, *gata5*, *gata6*, *tbx20*, and *tbx5*), apoptosis (*bcl2* and *casp9*), and inflammation response (*il6*). The formation of the cardiogenic region during early development is regulated by *gata4*, *gata5*, *gata6*, and *tbx5* [14]. Our findings display that AMX exposure altered the expression of these key regulators, suggesting interference with the molecular pathways required for early cardiac morphogenesis. This indicates that AMX can disrupt cardiovascular developmental processes, an effect that has not been comprehensively reported before. *tbx20* expression is another critical determinant of cardiomyocyte cell proliferation and lineage maturation during embryonic development, and it also contributes to heart regeneration. Our results indicate that AMX exposure caused a slight upregulation of *tbx20* at C1 (0.011 mg/L), though this was not statistically significant (*p* > 0.05). Thus, AMX does not appear to exert measurable effects on *tbx20* expression under the tested conditions. These findings are partly consistent with [22], who reported no significant effects of AMX on *gata6*, *hhex*, or *foxa3* expression after 5 days of exposure, suggesting limited impact on developmental pathways at environmentally relevant concentrations.

Apoptosis-related genes were more strongly affected. Anti-apoptotic proteins such as *bcl2* regulate and mediate the balance between survival and apoptosis in cells [23]. In the present study, AMX exposure significantly affected *bcl2* expression. Decreased bcl2 levels were observed at C1 exposure concentration (0.011 mg/L) suggesting potential pro-apoptotic effects of AMX at environmentally relevant concentrations. *casp9*, a gene from the caspase family, functions as an initiator of apoptosis [24]. Our results show that treatment groups C2 and C3 exhibited upregulation of *casp9* compared to controls, while C1 showed similar expression to controls. The combination of suppressed *bcl2* and increased *casp9* suggests a shift toward pro-apoptotic signaling, although further validation is needed to confirm activation of cell death pathways.

The inflammatory pathway also appeared sensitive to AMX exposure. IL-6, a major regulator of inflammation and tissue homeostasis, was significantly modulated across treatment groups. The gene was downregulated in C1 and C2 but upregulated in C3. This biphasic regulation suggests that AMX disrupts inflammatory signaling, which may act in synergy with developmental and apoptotic disturbances to impair cardiac function.

Taken together, these molecular findings provide mechanistic insight into how AMX may induce cardiotoxic effects in zebrafish embryos. Our findings align with [17], who observed delayed development and biochemical alterations following AMX exposure, although their study did not specifically assess cardiac endpoints. Similarly, ref. [18] reported genotoxic effects of AMX, reinforcing its broader toxic potential. In contrast, ref. [22] found only modest changes in *gata6*, consistent with our observation that not all developmental genes are equally affected. Overall, these comparisons suggest that AMX impacts developmental, apoptotic, and inflammatory pathways in zebrafish embryos, with cardiotoxic outcomes that appear to be both concentration- and endpoint-specific.

*AMX affected the heart function.* AMX had a significant effect on heart function in Zebrafish embryos, as by analyses of the PCV parameters. Cardinal veins are some of the first vessels to form during embryonic development. In our study, heart function parameters were the primary metrics used to assess AMX’s cardiotoxicity. Our findings revealed that exposure to AMX caused significant changes in blood flow and linear velocity within the PCV of AMX-treated embryos. Specifically, AMX exposure significantly affected the blood flow and linear velocity in the PCV at concentrations C2 and C3, with the greatest effect at 100 mg/L (treated group C3). The observed changes in gene expression, particularly the upregulation of *tbx20*, may contribute to compensatory responses in cardiac development in the PCV. However, despite significant changes in the blood flow and linear velocity, the relatively modest changes in the arterial pulse suggest that the physiological effects of AMX may require a longer observation period or higher concentrations to fully manifest. A study by [22] on antibiotic exposure found that AMX at certain concentrations had limited significant effects on genes related to early heart development but suggested potential long-term effects on cardiovascular health. These findings highlight the importance of using comprehensive heart function assessments to evaluate drug-induced cardiovascular changes in zebrafish models. The observed cardiovascular disturbances may be associated with pericardial edema, a hypothesis that requires further investigation. Prior research has shown that AMX along with other antibiotics can influence cardiovascular parameters [22], but the specific effects on early embryonic heart development and function observed in our study highlight the need for more detailed analysis and longer exposure times to fully understand AMX’s cardiotoxic potential.

This study has some limitations. First, we only evaluated short-term (96 hpf) exposures, and therefore potential long-term or multi-generational effects remain unknown. Second, although gene expression markers provide useful insights, some conclusions remain tentative. For example, the downregulation of *bcl2* alone cannot be considered definitive evidence of apoptosis without supporting assays, such as caspase activity or TUNEL staining. Finally, while this study focused on zebrafish, there are potential implications for human health. Given that AMX residues are frequently detected in wastewater and surface waters, bioaccumulation in aquatic organisms could represent a route of exposure through the food chain, warranting further ecotoxicological and epidemiological investigations.

## 4. Materials and Methods

### 4.1. Chemicals and Reagents

In this study, Amoxicillin trihydrate (AMX; Sigma Aldrich, cat#A8523, Burlington, MA, USA) was used. The medium “egg water” used for the growth of zebrafish embryos was prepared by mixing the following components: 17.53 g of 5.0 mM Sodium Chloride (NaCl), 0.76 g of 0.17 mM Potassium Chloride (KCL), 2.37 g of 0.6 mM Magnesium Sulfate (MgSO_4_). 7H20, 3.53 g of 0.4 mM Calcium Chloride-H_2_O (CaCl_2_-H_2_O), and 1 L of deionized distilled MilliQ water (ddH_2_O). The selected AMX concentrations were determined based on amounts measured in the environment (C1), sewage (C2), and acute exposures (C3). PureLink^TM^ RNA Mini Kit (cat#12183025), SuperScript^TM^ IV VILOTM Master Mix (cat#11756050), and SYBR Green SuperMix (cat#1725271) were purchased from Thermo Fisher Scientific (Waltham, MA, USA), Invitrogen (Waltham, MA, USA), and BioRad (Hercules, CA, USA), respectively.

### 4.2. Zebrafish Maintenance and Exposure

Wild-type adult zebrafish (AB strain) were maintained in the zebrafish facility within Qatar University’s Biomedical Research Center under strictly controlled laboratory conditions. These conditions included a consistent photoperiod of 14 h of light and 10 h of darkness, along with a constant water temperature of 28 °C and pH of 7.0. All husbandry and spawning procedures followed the protocols approved by Qatar University’s Animal Care and Use Committee (QU-IACUC). Since zebrafish embryos used in the present work were no more than 96 hpf, approval from the Institutional Biosafety Committee (IBC) for the use of zebrafish embryos was deemed sufficient (Approval ID: QU-IBC-2023/041). Following natural spawning, fertilized embryos (survival rate > 95%) were selected and exposed to AMX starting at 2 hpf. Embryos were randomly assigned to treatment groups to minimize selection bias. The AMX treatment concentrations were set at three levels: (C1) 0.011 mg/L; (C2) 18.68 mg/L; and (C3) 100 mg/L, with a negative control (0 mg/L) utilizing AMX-free egg water. Amoxicillin trihydrate (molecular weight: 419.45 g/mol; Sigma-Aldrich, Cat. PHR1127, see SDS) was dissolved in dimethyl sulfoxide (DMSO) to prepare stock solutions. Working concentrations were calculated by molar conversion and dilution into embryo medium. The selected exposure levels were based on literature values: C1 (11.99 µg/L; ~2.9 × 10^−8^ M) corresponds to the highest concentrations reported in surface waters and wastewater effluents [7]; C2 (18,680 µg/L; 18.68 mg/L; ~4.5 × 10^−5^ M) reflects concentrations detected in hospital/industrial sewage [4]; and C3 (100,000 µg/L; 100 mg/L; ~2.4 × 10^−4^ M) mimics acute exposure conditions used in zebrafish toxicity assays [17]. In six-well plates, 105 zebrafish embryos were divided into three groups, with each group containing 35 embryos. Within each group, triplicate experiments were conducted, resulting in three replicates for each selected concentration. In each well plate, 3 mL of media containing AMX at varying concentrations was used to house 11–12 zebrafish embryos per well. The experiment was conducted over 96 h post-fertilization (hpf), during which, half of the exposure solution was replaced daily to maintain stable contaminant concentrations. Every 24 h, measurements of the Mortality Rate (MR) and Hatching Rate (HR) were taken, and any deceased embryos were immediately removed from the wells to prevent interference.

### 4.3. Embryotoxicity Assessment

The embryotoxicity test was assessed every 24 h under a ZEISS stereomicroscope by calculating the percentages of mortality and hatching rates (%) in AMX-exposed fish.

### 4.4. RNA Isolation and qPCR Analysis

After treatment with AMX at different exposure concentrations, 96 hpf embryos were euthanized by an overdose of MS-222 (0.3%). Samples were preserved in RNAlater and stored at 4 °C until RNA extraction. For each exposure concentration, 20 embryos were pooled due to the small size of zebrafish embryos. This approach was taken to ensure adequate RNA quantities for experimentation. Total RNA was extracted using a PureLink^TM^ RNA Mini Kit (cat#12183025) following the manufacturer’s instructions. RNA concentration was determined at 260 nm using a NanoDrop (Thermo Scientific) and RNA samples with a ratio of absorbance (260/280 nm) ranging from 1.8 to 2.0 were considered suitable for cDNA synthesis. 350 ng of RNA was reverse transcribed into cDNA using SuperScript™ IV VILOTM Master Mix. Real-time quantitative PCR (RT-qPCR) was performed using a QuantStudio 6 Flex system (Applied Biosystems, Waltham, MA, USA) with SYBR Green SuperMix. The standard cycling conditions for all the target genes were 50 °C for 2 min, followed by 40 cycles of 95 °C for 15 s and 60 °C for 1 min. The melt curve stage conditions were 95 °C for 15 s and 60 °C for 15 s. Relative expression of target gene mRNA was determined by the ΔΔCt method, normalized to *elf1a* levels. The primer sequences for the target genes are listed in Table 1. Each qPCR assay was conducted in triplicate technical replicates to ensure reproducibility.

### 4.5. Heart Function Analysis

At 96 hpf, heart function analysis was conducted on nine embryos per treatment group, and the results were compared to controls. The analysis employed Micro ZebraLab Blood Flow software from Viewpoint (version 3.4.4, Lyon, France), following the protocol optimized by [25]. High-speed time-lapse recordings of the tail were captured using 1000 frames per 10 s under 100× magnification to measure the cardiac parameters in the posterior cardinal vein (PCV) including blood flow, arterial pulse, and linear velocity. The PCV was selected as it provides a large, optically accessible vessel for reliable quantification of hemodynamic parameters in zebrafish embryos [25]. Although other vessels (e.g., dorsal aorta) could provide complementary data, focusing on the PCV ensured consistency and reduced variability.

### 4.6. Statistical Analysis

For all the experiments conducted in the present study, statistical analysis was performed using GraphPad Prism 10 software using Dunnett’s test after One-Way ANOVA. The Shapiro–Wilk test was performed to test the normality of the data. Results are represented as Mean ± Standard Deviation; *p* < 0.05 was considered significant (*), *p* < 0.01 was considered highly significant (**), and *p* < 0.001 was considered very highly significant (***).

## 5. Conclusions

At the environmentally relevant concentration (0.011 mg/L), AMX modulated the expression of cardiac developmental genes (*gata5* and *gata6*) without causing significant alterations in heart function. At higher concentrations, corresponding to sewage treatment effluents (18.68 mg/L) and acute experimental exposure (100 mg/L), AMX significantly impaired blood flow and linear velocity and upregulated the apoptosis-related gene *casp9*, confirming clear cardiotoxicity. These results demonstrate that AMX affects zebrafish embryos in a concentration-dependent manner, linking subtle molecular disruptions at low doses to functional impairments at higher doses. Our work provides mechanistic insight into the ecotoxicological risks of antibiotic residues and highlights the potential hazards of pharmaceutical contaminants in aquatic environments. These findings underscore the need for stringent environmental monitoring and improved antibiotic residue management. Future studies should investigate long-term and multigenerational exposures and evaluate ecological consequences across aquatic species to better define risks of pharmaceutical contamination and guide wastewater management strategies.

## Figures and Tables

**Table 1:** Primer pairs of target genes in RT-qPCR analysis.

Genes	Primers	Sequence
*gata4*	Forward Reverse	5′-CCAGTCTGCAACGCATGTG-3′ 5′-GATCGCCGACTGACCTTCAG-3′
*gata5*	Forward Reverse	5′-GGGACGCCAGGGAACTCTA-3′ 5′-CACGCGTTGCACAGGTAGTG-3′
*gata6*	Forward Reverse	5′-AGTCGCGACCAGTACCTTTCAA-3′ 5′-CCTTCGGGATTGCAGTGAGT-3′
*tbx5*	Forward Reverse	5′-CGGATGTTTCCCAGCTTCAA-3′ 5′-CATCGCAGGCTCAGCTTTC-3′
*tbx20*	Forward Reverse	5′-GCACTCATGTCAAGTGGGAA -3′ 5′-CGAGGTTTGGATGGCATGA-3′
*bcl2*	Forward Reverse	5′-TCACTCGTTCAGACCCTCAT-3′ 5′-ACGCTTTCCACGCACAT-3′
*casp9*	Forward Reverse	5′-AAATACATAGCAAGGCAACC-3′ 5′-CACAGGGAATCAAGAAAGG-3′
*il6*	Forward Reverse	5′-TCAACTTCTCCAGCGTGATG-3′ 5′-TCTTTCCCTCTTTTCCTCCTG-3′

## Data Availability

The data presented in this study are openly available in the journal “Antibiotics”.

## Abbreviations

**Abbreviation**	**Full Term**
AMX	Amoxicillin
hpf	Hours Post-Fertilization
PCV	Posterior Cardinal Vein
DMSO	Dimethyl Sulfoxide
qPCR	Quantitative Polymerase Chain Reaction
LC50	Lethal Concentration 50%
LC18	Lethal Concentration 18%
SD	Standard Deviation
ANOVA	Analysis of Variance
EOCs	Emerging Organic Contaminants
EF1α	Elongation Factor 1-Alpha (housekeeping gene)
